# Utilization of acute medical services in general practice: a retrospective routine data analysis

**DOI:** 10.1186/s12245-025-00943-y

**Published:** 2025-08-07

**Authors:** Christoph Strumann, Wolfgang C. G. von Meißner, Paul-Georg Blickle, Johannes Rieken, Jost Steinhäuser

**Affiliations:** 1 Institute of Family Medicine, University Medical Center Lübeck, Ratzeburger Allee 160, Lübeck, 23562 Germany; 2 Hausärzte am Spritzenhaus, Freudenstädter Str. 36, Baiersbronn, 72270 Germany; 3 Europäische Fachhochschule, Konrad-Adenauer-Str. 25, Köln, 50996 Germany

**Keywords:** Acute care, Primary care, Emergency care, Outpatient services

## Abstract

**Background:**

The increasing utilization of emergency departments by patients with acute but non-emergency medical needs contributes to overcrowding in emergency care. Previous research has mainly focused on hospitals and out-of-hours care centres. The role of general practitioners providing primary care during office hours for emergency and acutely ill patients has not yet been considered intensively. This analysis aimed to quantify and describe the documented outpatient utilization behaviour of patients with acute care needs in primary care practices during office hours.

**Methods:**

The retrospective cohort study used routine data from 16 German primary care practices in 2022 and 2023 from the Supraregional Health Service Research Network. Acute care cases were identified as consultations without a prior appointment or those with a same-day appointment. Statistical analyses included bivariate and multivariate analyses.

**Results:**

A total of 873,732 consultations involving 90,020 patients were analysed. When considering only the first visit of an acute episode, 60.6% of cases were classified as acute. Patients seeking acute care were younger (51.9 vs. 58.3 years, *p* < 0.001) and more likely to visit the practice on Mondays (Odds Ratio: 1.48, *p* < 0.001) or at the weekend (Odds Ratio: 13.91, *p* < 0.001). Nonspecific health factors, respiratory-, musculoskeletal- and cardiovascular reasons for encounter dominated. The majority of acute cases (80%) did not seek any further health service on the same day, while approximately 19% of patients were referred to a specialist and 3% were admitted to a hospital within 14 days.

**Conclusion:**

The effective management of acute cases by primary care practices highlights the potential for strengthening this sector to enhance the quality and efficiency of emergency care.

**Supplementary Information:**

The online version contains supplementary material available at 10.1186/s12245-025-00943-y.

## Introduction

In many Organization for Economic Co-operation and Development (OECD) countries the demand for emergency care (both inpatient and outpatient) has increased over recent decades [1, 2]. A key factor contributing to the overcrowding of emergency departments (ED) is the rising number of patients seeking acute but non-emergency medical care in these facilities [3], which in turn impedes the effectiveness of emergency care [4, 5]. For instance, research indicates that a substantial proportion of all ED visits are non-urgent (e.g., Belgium: 56% [6], US: 30% [7], Germany: 27% [8], England: 15% [9]).

Beyond the ED, primary care serves by definition as the first point of contact for most patients addressing a broad spectrum of health concerns including acute medical conditions [10]. To ensure high-quality and continuity of primary care during out-of-hours periods, various health care models have been implemented, differing significantly between health care systems in terms of coordination, e.g., see [11] for Europe. Here the organization of the out-of-hours primary care models encompass single GPs taking care of their own patients 24 h a day, 7 days a week (e.g., Bulgaria, Malta, Romania), smaller or larger groups of GP cooperatives (e.g., Belgium, Denmark, Germany), primary care centers (Italy, Luxembourg, Poland, Sweden) and integrated primary care in hospitals (e.g., Finland). In general, less coordinated and fragmented health care systems tend to allocate scarce resources inefficiently and risk providing lower quality care [12].

Germany, for example, has a rather fragmented health care system in which patients with acute medical conditions can freely choose to enter the system through three distinct entry points, i.e., primary care practices during regular hours, out-of-hours primary care cooperatives or hospital EDs [13]. Changing patient behaviour coupled with inefficiencies in both emergency and primary care management have led to an suboptimal resource allocation [14]. On the one hand, there has been an increase in the use of EDs by patients with acute but non-urgent medical needs, while on the other hand, the number of out-of-hours primary care cases has declined [15]. Studies exploring the motives of ED attendees in Germany have identified various factors, including misjudged urgency on the part of patients and a lack of knowledge of alternative services [16–18], preference for treatment by a specialist [19], and unwillingness to use the primary care physician as their treatment coordinator [20].

A more centrally coordinated system is generally considered a potential solution to optimize the emergency care system [21]. However, to develop such a system, a comprehensive understanding of the entire health care framework—including both primary and secondary care—is essential. Most existing (international) research focuses on hospitals and their management of ED infrastructure [2, 3, 22–24]. Outpatient care has so far only been taken into account by considering the integration of out-of-hours primary care and ED services, e.g [1, 14, 15, 25–27]. The role of the outpatient sector as a whole in the treatment of emergency patients and patients with acute medical needs has not yet been considered [28]. The lack of evidence has resulted in an underrepresentation of this sector in the assessment of acute medical cases. To achieve a comprehensive evaluation of patient care, it is essential that all sectors involved are given due consideration. This comprehensive approach will facilitate the identification of any necessary structural adjustments, thereby ensuring the development of an efficient and effective solution.

The objective of the present study is to quantify and describe the outpatient utilization behaviour of patients with an urgent need for treatment in primary care practices in Germany. The aim of this retrospective data analysis is to ascertain the contribution of outpatient primary care physicians to the care of patients with acute consultation needs.

## Methods

### Study design and data collection

In this retrospective cohort study, routine data were used from 16 primary care practices of the Supraregional Health Service Research Network (SHRN), Germany [29] covering the full years 2022 and 2023, i.e., from January 1, 2022, to December 31, 2023. The SHRN is a collaboration of the Institute of Family Medicine in Lübeck and private practices that routinely provide anonymized electronic health record (EHR) data for research purposes. The data collection infrastructure includes a standardized, practice-level software interface that extracts consultation-level information (e.g., appointment type, ICD-10 diagnosis, referral, and incapacity certification) directly from the practices’ EHR systems. This process is automated, so no additional documentation from physicians is required (see [29] for more details about the data processing).

### Study population

The participating practices are located in the federal state of Baden-Württemberg in southwestern Germany, encompassing both urban and rural areas. All have an appointment system through which patients can make appointments in various ways (by telephone, online, or in person). The practices are located in close proximity to hospitals with open-access EDs, a typical feature of the German health care landscape. In Germany, 80% of the population can drive to the nearest emergency room or ambulance in less than 15 min, 17% in less than 24 min, while the remaining 3% need more than 24 min [30]. Primary care is provided mainly by office-based general practitioners (GPs) who operate independently and serve as the main point of entry into the health care system during regular office hours (7 a.m. to 6 p.m.). There is no requirement for patients to register with a specific GP, nor is there any gatekeeping system; patients are free to directly access specialist care or EDs [31]. Further, since 2019 GPs are obliged to offer at least 5 h per week a flexible scheduling without an appointment [32].

All patients with at least one documented consultation in one of the participating practices during the study period (2022–2023) were included in the analysis, without restrictions regarding age, sex, or insurance type. The dataset includes data from more than 90,000 different patients and represents a comprehensive sample of over 850,000 routine primary care encounters across all patient groups and, thus, reflects the actual utilization patterns observed in German primary care settings. Due to high morbidity and an ageing German population, the average number of consultations per patient per year (4.7) is in line with other figures that highlight the high demand for health care in Germany [33], even though visits to the doctor tend to be relatively short [34].

### Study variables

Consultations for acute care cases were identified as visits without a previous appointment or with an appointment made on the same day as the visit. These cases were considered as an urgent need for treatment in primary care, irrespective of whether they were classified as emergency or non-emergency cases, since it was not possible to differentiate between these categories. As our definition of acute cases might lead to a rather liberal definition of acuteness, control visits after the initial visit were also taken into account. Therefore, a second (conservative) definition is applied by considering only the first visits of an acute episode, where an episode is defined as having ended if the patient has not returned to the practice within 14 days of the last consultation. One practice of the original 17 primary care practices of the SHRN was not considered, since appointments were not documented in the electronic health records. As an outcome variable, the course of the episode of care is considered. We differentiate between (1) no further treatment needed, (2) further treatment solely in the primary care practices, (3) referral to a specialist (outpatient care) and (4) hospitalization (inpatient care). In the German health care system, secondary care is provided either as outpatient specialist treatment (e.g., by dermatologists or orthopaedists) or as inpatient treatment in hospitals. The time for progression is considered for different time horizons, i.e., same day, 3 days and 2 weeks.

Other variables include case characteristics, such as the weekday of the date of the consultation, the documented diagnoses, and whether a certificate of incapacity for work was issued, as well as patient characteristics, such as the patient’s age, gender, and insurance status on the date of the consultation.

In Germany, all employed citizens, as well as other groups such as pensioners and those earning less than the opt-out threshold (€66,600 per year in 2023 [35]), are legally required to have statutory health insurance. The insurance for their non-earning dependants is provided free of charge. Individuals whose gross income exceeds the threshold, in addition to those who are self-employed, have the option to either maintain their statutory health insurance on a voluntary basis or to purchase private health insurance. Approximately 87% of the population are statutorily, while 11% are privately insured [36].

### Statistical analysis

To quantify and describe the outpatient utilization behaviour of patients with an urgent need, the statistical analyses were conducted on the consultation level. First, we compute the frequencies for acute and non-acute (elective) cases. Second, bivariate analyses were applied to describe the patient and consultation characteristics of acute and elective cases. Differences between both subgroups were tested for statistical significance using either the Pearson $\:{\chi\:}^{2}$-test or t-test, depending on the scale of the variable considered. Third, multivariate multilevel (ordered) logistic regression analyses were used to identify key determinants of acute care episodes and structural differences between acute and elective cases, and different progressions of the episodes among acute cases. Here, only the first visits of the episodes were considered. The following variables were considered as covariates: categories of the patient’s age (to account for nonlinear effects), gender, insurance status, whether a certificate of incapacity for work was issued, the weekday, and diagnosis chapters. Further, individual effects were specified on the patient and practice level.

In the bivariate analysis, missing values were deleted of casewise, while in the multivariate analysis, they were deleted listwise.

## Results

### Sample characteristics

In total, 873,732 consultations were documented in the 16 primary care practices in 2022 and 2023. Of these consultations, 623,162 (71.3%) were identified as acute, i.e., visits without a previous appointment or with a same-day appointment (see Table 1). Regarding only the first visit of an episode (i.e., acute cases without a previous visit to the practice within 14 days), 385,487 of 636,057 first visit consultations (60.6%) were observed. The number of consultations with an appointment made prior to the same day (elective, *n* = 250,570), remained constant if only the initial visit was taken into consideration.

**Table 1:** Sample characteristics

		Acute	Elective	Odds-Ratio
Consultations		*n* = 623,162 (71.3%)	*n* = 250,570 (28.7%)	
		CI95%: [71.2;71.4]	CI95%: [28.6;28.8]	
Age, µ (SD)		54.5(22.5)	58.3 (19.6)	0.99**
Female, *n* (%)		348,103 (55.9)	138,699 (55.4)	1.02**
Male, *n* (%)		273,761 (43.9)	111,793 (44.6)	0.97**
Statutorily insured, *n* (%)		594,643 (95.4)	233,694 (93.3)	1.51**
Privately insured, *n* (%)		25,766 (4.1)	16,525 (6.6)	0.61**
Certificate of incapacity for work (18-65-year-olds), *n*/*N* (%)		119,380/372,136 (32.1)	24,354/141,286 (17.2)	2.27**
*Most frequent diagnoses*				
Z00-Z99	Factors influencing health status and contact with health services	117,065 (18.8)	62,863 (25.1)	0.69**
J00-J99	Diseases of the respiratory system	85,819 (13.8)	16,736 (6.7)	2.23**
U00-U99	Codes for special purposes	47,457 (7.6)	7,359 (2.9)	2.72**
First visit of an episode		*n* = 385,487 (60.6%)	*n* = 250,570 (39.4%)	
		CI95%: [60.5;60.7]	CI95%: [39.3;39.5]	
Age, µ (SD)		51.9 (22.0)	58.3 (19.6)	0.99**
Female, *n* (%)		211,554 (54.9)	138,699 (55.4)	0.98**
Male, *n* (%)		173,075 (44.9)	111,793 (44.6)	1.01**
Statutorily insured, *n* (%)		367,797 (95.4)	233,694(93.3)	1.50**
Privately insured, *n* (%)		17,040 (4.4)	16,525(6.6)	0.66**
Certificate of incapacity for work (18-65-year-olds), *n*/*N* (%)		71,588/245,974 (29.1)	24,354/141,286 (17.2)	1.97**
*Most frequent diagnoses*				
Z00-Z99	Factors influencing health status and contact with health services	88,793 (23.0)	62,863 (25.1)	0.89**
J00-J99	Diseases of the respiratory system	59,434 (15.4)	16,736 (6.7)	2.55**
R00-R99	Symptoms, signs and abnormal clinical and laboratory findings	29,533 (7.7)	26,020 (10.4)	0.63**

90,020 patients with 873,732 consultations and 636,057 first visits, i.e., cases without a previous visit to the practice within 14 days

*p < 0.01

**p < 0.001

The mean age of patients seeking for acute care is about four years lower than that of patients with an elective appointment (54.5 vs. 58.3, *p* < 0.001). Considering only the first visit, this difference increases to more than six years. The percentage of females were slightly higher in the group of patients requiring acute care, while it was lower if only the first visit of an episode was taken into account. Approximately 60% of the consultations were attended by patients within the main working-age group (18–65 years). In 32.1% of acute cases, a certificate of incapacity for work was documented, in comparison with 17.2% of cases within an elective consultation.

**Fig. 1:**
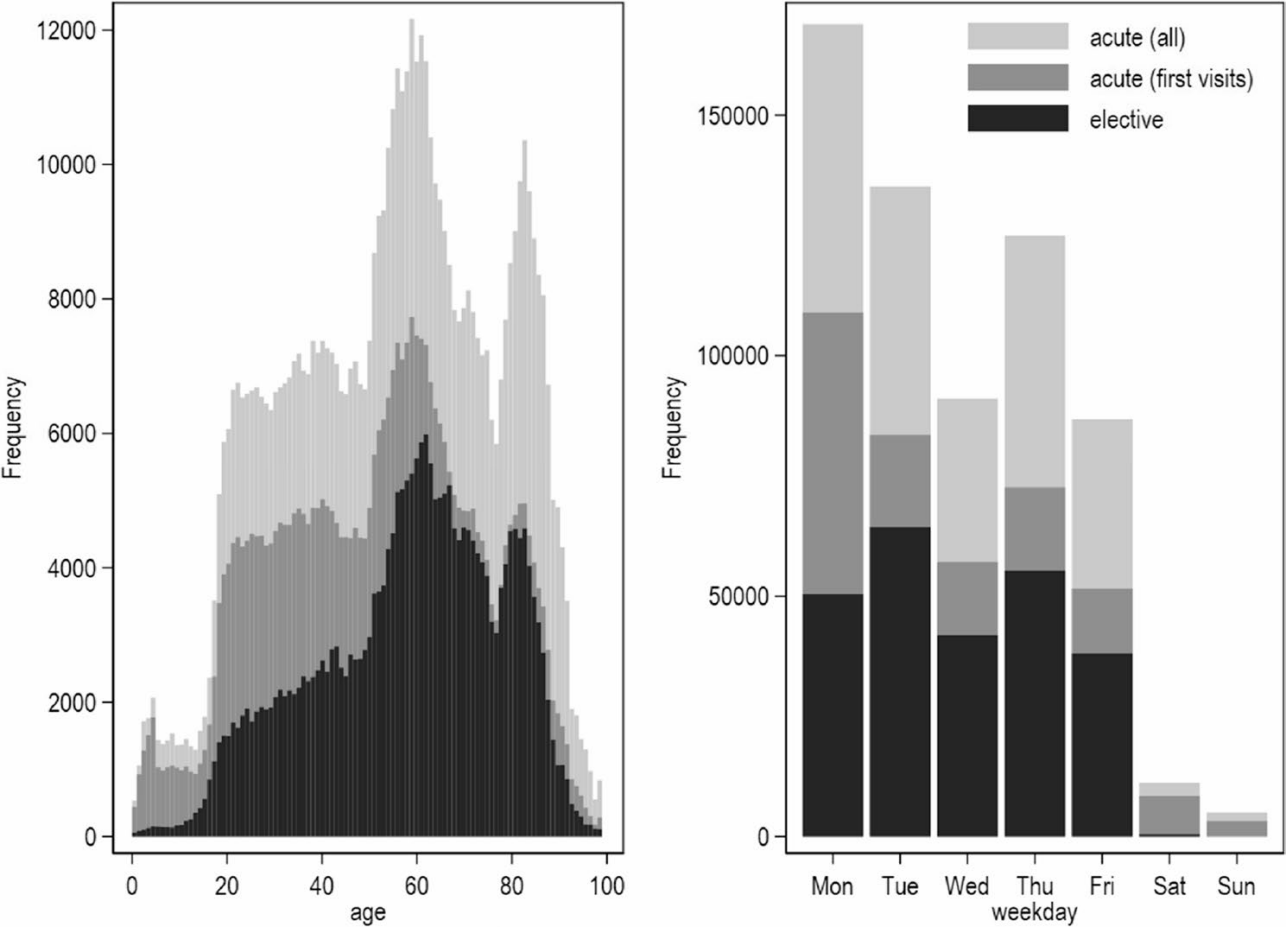
Age and weekday distribution

Figure 1 shows the age (left panel) and weekday distribution (right panel) for all acute cases, for the first visit of an episode of acute cases and for elective cases. From ages 20 to 65, the proportion of each type of case remains relatively stable and increases over time. For example, at age 20, there were approximately 1,500 elective visits, 4,000 acute first visits, and 6,500 acute consultations. These numbers increase proportionally with age until age 65. At this age, the number of acute first visits decreases relatively to the other types of cases. Thus, for patients over 65 years of age, the proportion of acute first visits is markedly lower compared to that of younger patients. The distributions over the weekdays indicates Monday to be the day with the highest number of consultations and with the highest percentage of acute cases (all consultations: 77%, first visits: 68%), except for the weekend, where almost all cases were acute.

Differences between the acute care consultations that go along without a previous appointment and with a same-day appointment are shown in Table S1 in the supplements. Almost 20% of patients visiting the practice for acute care made an appointment on the same day. On average, these patients were 9 years younger, and a certificate of incapacity for work was documented in 57.0% of cases.

### Diagnoses

The frequencies of acute and elective cases of the first visits for the three most frequent diagnoses based on the ICD10 classification were also shown in Table 1. A full list of all diagnoses is provided in Table S2 and Table S3 in the supplements. A non-specific Z-diagnosis (“Factors influencing health status and contact with health services”) was documented in nearly one out of every four cases, with a higher likelihood in elective cases (Odds Ratio [OR] 0.89, *p* < 0.001). The second most common diagnosis among the acute cases belongs to the diseases of the respiratory system (J00-J99). The percentage is significantly larger than in the group of the elective cases (OR, 2.55. *p* < 0.001).

### Outcome

In Table 2, the percentages of the acute cases are shown for different courses of the episode within the same day, 3 days and 2 weeks. We differentiate whether the patients have visited only a primary care practice or received only a referral to specialist.

**Table 2:** Course of the episode of care, *n* (%)

within	No additional health service consumption besides practice	Visit in same practice	Referral to specialist	Hospitalization
Same day^a^	308,083 (79.9)	-	73,707 (19.1)	9,742 (2.5)
Only^b^		-	67,662 (17.6)	
3 days^a^	267,849 (69.5)	52,266 (13.6)	79,525 (20.6)	11,020 (2.9)
Only^b^		34,241 (8.9)	72,377 (18.8)	
2 weeks^a^	210,977 (54.7)	124,482 (32.3)	91,525 (23.7)	13,046 (3.4)
Only^b^		78,845 (20.5)	82,619 (21.4)	

90,020 patients with 636,057 first visits, i.e., cases without a previous visit to the practice within 14 days

^a^The course of the episode of care is considered within the same-day, 3 days, or 2 weeks

^b^Patients have either visited a primary care practice or received a referral to a specialist, but nothing else

For 308,083 (79.9%) of the consultations, the patients did not seek any further health service consumption at the same day, while in 19.1% and 2.5% of the consultations, the patients were referred to a specialist and/or hospital, respectively. After two weeks, 54.7% of consultations resulted in patients not seeking any health services. For one third of the consultations, patients have visited a primary care practice. Patients were referred to a specialist in 23.7% of cases and hospitalized in 3.4% of cases. The percentage of cases with patients who have visited a primary care practice only after two weeks is 20.5%, indicating that in over 75% of the cases, patients did not require any further care outside primary care practices after two weeks of their initial visit for acute care.

### Multivariate analysis

Table 3 shows the estimated ORs of the multilevel logistic regression models for the identification of acute cases and the course of the episode of care of acute cases. After controlling for individual effects on the patient and practice level as well as for the diagnosis of the case, the sex of the patients did not affect the likelihood of an acute case. However, women with an acute care need were less likely to have a severe progression of their episode of care. This difference becomes smaller the longer the period considered for the course of the episode is (OR: 0.90, *p* < 0.001 for same-day and OR: 0.98, *p* < 0.01 for 2 weeks). The age of the patient has an influence on both, the likelihood of the case being acute and the course of the episode of care. While the former decreases with age, the latter increases, i.e., younger patients are more likely to present with an acute need for care and less likely to have a severe progression of their episode of care. The highest likelihood for a severe progression is observed for patients aged between 50 and 64. A certificate of incapacity for work is associated with a higher chance of an acute case, but reduces the likelihood for a severe progression. Patients visiting the practice on Mondays are more likely to have an acute episode and more likely to have a severe episode. Weekends are associated with the highest probability of an acute episode and the lowest probability of a severe progression.

**Table 3:** Multilevel regression results

Odds Ratios	acute care at first visit	course of the episode of care		
		same-day	3 days	2 weeks
model	Logit (1: acute, 0: elective)	Ordered Logit (1: no additional health service consumption, 2: visit in practice, 3: referral to specialist, 4: hospitalization)		
female	0.99	0.90**	0.93**	0.98*
age (years, reference: *0–6*)				
* 7–17*	0.52**	1.40**	1.57**	1.69**
* 18–29*	0.40**	1.70**	1.93**	2.08**
* 30–49*	0.34**	1.76**	1.91**	2.21**
* 50–64*	0.27**	3.15**	2.93**	3.49**
* 65–80*	0.22**	1.91**	2.08**	2.90**
* > 80*	0.27**	1.31**	1.69**	2.85**
statutorily insured	1.60**	1.46**	1.40**	1.41**
certificate of incapacity for work	1.93**	0.52**	0.64**	0.82**
weekdays (reference: Monday)				
* Tuesday*	0.57**	0.91**	0.86**	0.93**
* Wednesday*	0.60**	0.80**	0.71**	0.85**
* Thursday*	0.57**	0.86**	0.61**	0.91**
* Friday*	0.59**	0.79**	0.66**	0.89**
* weekend*	9.61**	0.13**	0.26**	0.31**
observations	636,016	385,446	385,446	385,446
patients	90,015	83,626	83,626	83,626

Individual practice and patient level effects; estimated effects of dummy variables for ICD10-Chapters are suppressed; a full list with all regression variables with the 95% confidence intervals is provided in Supplements (Table S4); significance levels: ^*^p < 0.01, ^**^p < 0.001

## Discussion

The present study quantifies and describes the outpatient utilization behaviour of patients with an urgent need for treatment in primary care practices in Germany and aims to ascertain the contribution of outpatient primary care physicians to the care of patients with acute consultation needs. Our data of over 870,000 consultations reveal that at least 60% of all primary care consultations are for acute care needs, i.e., excluding spontaneous or informally scheduled control visits. Patients with acute problems were on average younger than those with scheduled appointments. The vast majority of patients (75%) did not require further treatment outside the primary care practice after an acute consultation.

This finding indicates that the vast majority of these cases can be effectively managed within primary care. In light of the observation that a considerable number of EDs are overcrowded due to an increase in patients seeking non-emergency, acute medical care [3], one might argue that the proportion of these patients would be even higher without the effective management in primary care. Therefore, primary care services appear to play a pivotal role in alleviating the burden of ED overcrowding. A recent study show that at weekends more non-urgent emergency attendances in German EDs were observed [22], suggesting that patients who would visit a primary care practice with their acute care needs directly visit a hospital’s ED when the practices are closed. There are several studies providing evidence for reducing demand for emergency care by increased primary care access [37, 38]. For instance, in the Netherlands, general practitioners are increasingly providing out-of-hours primary care on site in collaboration with hospital EDs, referring only 4% of self-referred patients to the ED [39]. In Australia, the opening of an after-hours general practice clinic has led to a reduction in low-acuity emergency department presentations [40]. Primary care practices that started to open every day a week in the UK reduced general ED attendances by 9.9% [41], and reduced minor problems and costs by more than a quarter [21]. In Italy, an extension of primary care availability reduced inappropriate ED admissions by 10 to 15% [42]. In Germany, the empirical evidence is rather scarce. However, ambulatory care-sensitive ED cases were found to be higher in districts with lower physician density [43], suggesting that EDs tend to compensate for the lack of primary care physicians especially in rural areas [44]. Recent approaches rely on expanding access to primary care through telemedicine services. Promising results being demonstrated by a direct-to-patient telemedicine service offered by primary care physicians for acute care in Southwestern Germany. In the pilot project called docdirekt, over 88% of the telemedical consultations were resolved and half of the patients indicated that they would otherwise have visited a hospital ED [45].

Our findings show that younger patients are more likely to seek acute primary care compared to older patients. One possible explanation for this is the requirement for a certificate of incapacity for work. Employees in Germany need this certificate as a legal prerequisite for income replacement schemes in the event of illness [46]. To obtain this certificate, employees usually were obliged to obtain it from a primary care practice [47]. This is consistent with the observation that patients of retiring age (over 65 years) have a lower proportion of acute first visits than younger patients, indicating that older patients visit the practice more frequently after an acute consultation than younger patients. The latter may visit the practice primarily to obtain a certificate of incapacity for work, while older patients may be more likely to require follow-up visits due to complex or chronic conditions that necessitate monitoring after an acute episode. Interestingly, our analysis suggests that patients aged between 50 and 64 have the highest risk of severe disease progression. This may indicate that this age group, while still part of the work-force, is increasingly affected by chronic conditions that can acutely deteriorate. For this patient group it might be of particular importance to establish a regular relationship with a primary care physician to foster continuity of care [48]. Regular appointments and coordination of services tailored to patients’ preferences has been demonstrated to enable better self-management and a rapid response to a deteriorating situation, thereby avoiding acute care visits [49].

The diagnosis distribution in our data indicates that non-specific health factors and respiratory diseases are the most common acute complaints. This aligns with previous research showing that respiratory infections are among the leading causes of demanding out-of-hours care [50]. In contrast to this finding are the results of a study investigating German ED attendances, where symptoms related to musculoskeletal/locomotor system skin, the digestive system and circulatory system as well as neurological symptoms were more often than respiratory symptoms [51]. Several studies indicate a high and increasing prevalence of acute admissions to EDs of patients with nonspecific diagnoses [26, 52, 53]. According to the ICD10, patients who are discharged from hospital without an established diagnosis should be recorded with a nonspecific diagnosis [54]. Nonspecific diagnoses can occur when there is a lack of progression of the disease and a high degree of diagnostic uncertainty [55, 56]. Dealing with diagnostic uncertainty due to ambiguous and undifferentiated symptoms are one of the core competences of primary care physicians [57]. Tolerance of uncertainty is associated with lower costs of investigation and treatment [58]. Patients with nonspecific diagnoses tend to have higher mortality rates since they often do not receive tailored therapy [59]. This may be due to the fact that patients discharged from the ED with a nonspecific diagnosis do not receive further monitoring or follow-up care, unlike those who initially consult a primary care physician providing “watchful waiting” and “continuity of care” [10]. Integrating this approach more effectively into acute care pathways could help reduce avoidable hospitalizations, reduce health care costs and improve patient outcomes.

### Strengths and limitations

While other studies have examined acute care demand by considering data from EDs or out-of-hours care centres, this is the first study to quantify and describe the outpatient utilization behaviour of patients with an urgent need for treatment from the perspective of primary care practices in Germany. By using real-world data on a large scale, we were able to encompass a substantial number of patients, drawn from 16 primary care practices in Germany. The data used provide unique insights into the management of patients seeking acute care demand. By using a pragmatic approach based on the practice appointment system rather than relying on the individual assessment of the treating physician, we were able to identify acute care needs from the patient’s perspective.

Despite these advantages, the use of real-world data has also some inherent weaknesses, most notably low internal validity. This applies especially to the operationalization of the acute care identification. Although all participating practices have a practice appointment system, we cannot guarantee whether the individual case is related to an acute care need or whether the appointment was made informally. Therefore, our estimated percentage of acute cases of 71.3% must be regarded as a liberal upper boundary. The consideration of only first visits of an acute episode offers a conservative lower bound estimate for the proportion of acute cases, i.e., 60.6%. Another limitation concerns the incomplete documentation of healthcare utilization outside the participating practices. Unreferred visits to other primary care providers, specialists, or emergency departments are only included in the dataset if they were retrospectively documented in the electronic health record, which may lead to underestimation of subsequent care. Although we were able to take into account the information of over 800,000 primary care consultations, the focus on only 16 practices constrains the representativeness of the findings. However, unlike routine data from statutory health insurance funds, the data from this study is not limited to statutorily-insured patients, which cover 84% of the population of Baden-Württemberg, where the practices are located [60]. The proportion of statutorily-insured patients in our sample (91.8%) is higher than the state average, which may be explained by the predominantly rural and semi-rural location of the participating practices. Studies indicate that private health insurance is less common in these regions, as it is more prevalent among higher-income individuals who are typically concentrated in urban areas [61]. Based on our results it would be possible to provide diagnostic specific estimates of the proportion of acute care enabling to classify cases into acute and elective care, similar as provided by Krämer et al. [62] for hospital admissions. This could be used to quantify the contribution of outpatient primary care physicians to the care of patients with acute consultation needs based on samples with a higher representativeness as e.g., invoicing data of the German Association of Statutory Health Insurance Physicians.

## Conclusion

The study highlighted the important role of primary care practices in managing acute medical cases, with over 60% of consultations classified as acute. The vast majority of these cases can be effectively managed in primary care, avoiding emergency department visits. These findings suggest that strengthening primary care services has the potential to improve emergency care efficiency and reduce unnecessary emergency department visits. Future research should focus on improving care coordination and access by exploring innovative approaches, such as telemedicine, to improve acute care management in primary care settings.

## Supplementary Information

Supplementary Material 1: Table S1.

Supplementary Material 2: Table S2.

Supplementary Material 3: Table S3.

Supplementary Material 4: Table S4.

Supplementary Material 5: STROBE Statement - checklist of items that should be included in reports of observational studies.

## Data Availability

The datasets generated and analysed during the current study are not publicly available due to strict German data protection regulations, the legal provisions with the Supraregional Health Service Research Network (SHRN) and the guidelines of the ethics committee of Luebeck University, but are available from the corresponding author on reasonable request.

## Abbreviations

ED: Emergency Departments
OECD: Organization for Economic Co-operation and Development
OR: Odds Ratio
SHRN: Supraregional Health Service Research Network
