# Supplementary material for: Utilization of acute medical services in general practice: a retrospective routine data analysis
Source: Int J Emerg Med. 2025 Aug 7;18:147. doi: 10.1186/s12245-025-00943-y (PMC12333268; doi:10.1186/s12245-025-00943-y)
Supplement: Supplementary file 1 — Supplementary Material 1: Table S1. [file 12245_2025_943_MOESM1_ESM.docx]

**Table S1. Sample Characteristics of patients with acute visits, n(%)**

|  | **Appointment**  **on same day** | **No appointment** | **Odds-Ratio** |
| --- | --- | --- | --- |
| **Consultations** | **n=120,106 (19.3%)** | **n=503,056 (80.7%)** |  |
| Age, µ (SD) | 47.0 (20.7) | 56.3 (22.5) | 0.98** |
| Female, n (%) | 64,240 (53.5) | 283,863 (56.4) | 0.89** |
| Male, n (%) | 55,816 (46.5) | 217,945 (43.3) | 1.14** |
| Statutorily insured, n (%) | 112,294(93.5) | 482,349 (95.9) | 0.62** |
| Privately insured, n (%) | 7,689 (6.4) | 18,077 (3.6) | 1.83** |
| Certificate of incapacity for work  (18-65-year-olds), n/N (%) | 51,030/89,530 (57.0) | 68,350/282,606 (24.2) | 4.15** |
| **First visit of an episode** | **n=82,870 (21.5%)** | **n=302,617 (78.5%)** |  |
| Age, µ (SD) | 44.7 (20.1) | 53.8 (22.0) | 0.98** |
| Female, n (%) | 43,681 (52.7) | 167,873 (55.5) | 0.89** |
| Male, n (%) | 39,140 (47.2) | 133,935 (44.3) | 1.13** |
| Statutorily insured, n (%) | 76,913 (92.8) | 290,884 (96.1) | 0.52** |
| Privately insured, n (%) | 5,844 (7.1) | 11,196 (3.7) | 1.97** |
| Certificate of incapacity for work  (18-65-year-olds), n/N (%) | 36,020/63,919 (56.4) | 35,568/182,055 (19.5) | 5.32** |

85,391 patients with 623,162 acute consultations and 385,487 acute first visits, i.e., cases without a previous visit to the practice within 14 days, **p* < 0.01, ***p* < 0.001
