# Supplementary material for: Utilization of acute medical services in general practice: a retrospective routine data analysis
Source: Int J Emerg Med. 2025 Aug 7;18:147. doi: 10.1186/s12245-025-00943-y (PMC12333268; doi:10.1186/s12245-025-00943-y)
Supplement: Supplementary file 2 — Supplementary Material 2: Table S2. [file 12245_2025_943_MOESM2_ESM.docx]

**Table S2. Diagnoses of all visits, n (%)**

| **ICD-Chapter** | **Description** | **acute** | **elective** | **Odds-Ratio** |
| --- | --- | --- | --- | --- |
| Z00-Z99 | Factors influencing health status and contact with health services | 117,065 (18.8) | 62,863 (25.1) | 0.69** |
|  | *subchapters* |  |  |  |
| Z00-Z13 | Persons encountering health services for examination and investigation | 44,640 (7.2) | 24,297 (9.7) | 0.72** |
| Z20-Z29 | Persons with potential health hazards related to communicable diseases | 11,312 (1.8) | 33,829 (13.5) | 0.12** |
| Z30-Z39 | Persons encountering health services in circumstances related to reproduction | 646 (0.1) | 124 (0) | 2.10** |
| Z40-Z54 | Persons encountering health services for specific procedures and health care | 1,824 (0.3) | 997 (0.4) | 0.73** |
| Z55-Z65 | Persons with potential health hazards related to socioeconomic and psychosocial circumstances | 714 (0.1) | 605 (0.2) | 0.47** |
| Z70-Z76 | Persons encountering health services in other circumstances | 56,233 (9) | 6,555 (2.6) | 3.69** |
| Z80-Z99 | Persons with potential health hazards related to family and personal history and certain conditions influencing health status | 5,667 (0.9) | 3,807 (1.5) | 0.59** |
| J00-J99 | Diseases of the respiratory system | 85,819 (13.8) | 16,736 (6.7) | 2.23** |
| U00-U99 | Codes for special purposes | 47,457 (7.6) | 7,359 (2.9) | 2.72** |
|  | *Covid related diagnoses* |  |  |  |
| U07.1 | COVID-19, virus identified | 28,291 (4.5) | 1,704 (0.7) | 6.95** |
| U07.2 | COVID-19, virus not identified | 2,559 (0.4) | 208 (0.1) | 4.96** |
| U08 | Personal history of COVID-19 | 908 (0.1) | 409 (0.2) | 0.89 |
| U09 | Post COVID-19 condition | 463 (0.1) | 706 (0.3) | 0.26** |
| U10 | Multisystem inflammatory syndrome associated with COVID-19 | 2 (0.0) | 4 (0.0) | 0.20 |
| U11 | Need for immunization against COVID-19 | 15,727 (2.5) | 6,497 (2.6) | 0.97 |
| U12 | COVID-19 vaccines causing adverse effects in therapeutic use | 186 (0.0) | 26 (0.0) | 2.88** |
| R00-R99 | Symptoms, signs and abnormal clinical and laboratory findings, not elsewhere classified | 45,261 (7.3) | 26,020 (10.4) | 0.68** |
| M00-M99 | Diseases of the musculoskeletal system and connective tissue | 40,340 (6.5) | 26,124 (10.4) | 0.59** |
| I00-I99 | Diseases of the circulatory system | 20,783 (3.3) | 16,555 (6.6) | 0.49** |
| A00-B99 | Certain infectious and parasitic diseases | 20,368 (3.3) | 5,756 (2.3) | 1.44** |
| S00-T98 | Injury, poisoning and certain other consequences of external causes | 16,363 (2.6) | 6,242 (2.5) | 1.06** |
| E00-E90 | Endocrine, nutritional and metabolic diseases | 15,649 (2.5) | 15,340 (6.1) | 0.40** |
| F00-F99 | Mental and behavioral disorders | 15,506 (2.5) | 12,213 (4.9) | 0.50** |
| G00-G99 | Diseases of the nervous system | 12,344 (2.0) | 7,478 (3.0) | 0.66** |
| K00-K93 | Diseases of the digestive system | 12,091 (1.9) | 8,572 (3.4) | 0.56** |
| N00-N99 | Diseases of the genitourinary system | 11,891 (1.9) | 5,984 (2.4) | 0.80** |
| L00-L99 | Diseases of the skin and subcutaneous tissue | 8,098 (1.3) | 5,793 (2.3) | 0.56** |
| C00-D48 | Neoplasms | 7,407 (1.2) | 4,193 (1.7) | 0.71** |
| H60-H95 | Diseases of the ear and mastoid process | 5,248 (0.8) | 2,318 (0.9) | 0.91** |
| H00-H59 | Diseases of the eye and adnexa | 4,098 (0.7) | 1,729 (0.7) | 0.95 |
| D50-D90 | Diseases of the blood and blood-forming organs and certain disorders involving the immune mechanism | 2,697 (0.4) | 2,227 (0.9) | 0.48** |
| Q00-Q99 | Congenital malformations, deformations and chromosomal abnormalities | 1,692 (0.3) | 934 (0.4) | 0.73** |
| O00-O99 | Pregnancy, childbirth and the puerperium | 400 (0.1) | 218 (0.1) | 0.74** |
| V01-Y84 | External causes of morbidity and mortality | 100 (0.0) | 92 (0.0) | 0.44** |
| P00-P96 | Certain conditions originating in the perinatal period | 23 (0.0) | 5 (0.0) | 1.85 |

90,020 patients with 873,732visits, **p* < 0.01, ***p* < 0.001
