# Supplementary material for: Utilization of acute medical services in general practice: a retrospective routine data analysis
Source: Int J Emerg Med. 2025 Aug 7;18:147. doi: 10.1186/s12245-025-00943-y (PMC12333268; doi:10.1186/s12245-025-00943-y)
Supplement: Supplementary file 3 — Supplementary Material 3: Table S3. [file 12245_2025_943_MOESM3_ESM.docx]

**Table S3. Diagnoses of first visits, n (%)**

| **ICD-Chapter** | **Description** | **acute** | **elective** | **Odds-Ratio** |
| --- | --- | --- | --- | --- |
| Z00-Z99 | Factors influencing health status and contact with health services | 88,793 (23.0) | 62,863 (25.1) | 0.89** |
|  | *subchapters* |  |  |  |
| Z00-Z13 | Persons encountering health services for examination and investigation | 28,986 (7.5) | 24,297 (9.7) | 0.76** |
| Z20-Z29 | Persons with potential health hazards related to communicable diseases | 7,162 (1.9) | 33,829 (13.5) | 0.12** |
| Z30-Z39 | Persons encountering health services in circumstances related to reproduction | 535 (0.1) | 124 (0) | 2.81** |
| Z40-Z54 | Persons encountering health services for specific procedures and health care | 758 (0.2) | 997 (0.4) | 0.49** |
| Z55-Z65 | Persons with potential health hazards related to socioeconomic and psychosocial circumstances | 440 (0.1) | 605 (0.2) | 0.47** |
| Z70-Z76 | Persons encountering health services in other circumstances | 50,475 (13.1) | 6,555 (2.6) | 5.61** |
| Z80-Z99 | Persons with potential health hazards related to family and personal history and certain conditions influencing health status | 3,303 (0.9) | 3,807 (1.5) | 0.56** |
| J00-J99 | Diseases of the respiratory system | 59,434 (15.4) | 16,736 (6.7) | 2.55** |
| R00-R99 | Symptoms, signs and abnormal clinical and laboratory findings, not elsewhere classified | 29,533 (7.7) | 26,020 (10.4) | 0.72** |
| M00-M99 | Diseases of the musculoskeletal system and connective tissue | 26,295 (6.8) | 26,124 (10.4) | 0.63** |
| U00-U99 | Codes for special purposes | 23,530 (6.1) | 7,359 (2.9) | 2.15** |
|  | *Covid related diagnoses* |  |  |  |
| U07.1 | COVID-19, virus identified | 9,535 (2.5) | 1,704 (0.7) | 3.70** |
| U07.2 | COVID-19, virus not identified | 1,456 (0.4) | 208 (0.1) | 4.56** |
| U08 | Personal history of COVID-19 | 610 (0.2) | 409 (0.2) | 0.97** |
| U09 | Post COVID-19 condition | 268 (0.1) | 706 (0.3) | 0.25** |
| U10 | Multisystem inflammatory syndrome associated with COVID-19 | 2 (0) | 4 (0) | 0.33** |
| U11 | Need for immunization against COVID-19 | 14,669 (3.8) | 6,497 (2.6) | 1.49** |
| U12 | COVID-19 vaccines causing adverse effects in therapeutic use | 56 (0) | 26 (0) | 1.40** |
| I00-I99 | Diseases of the circulatory system | 13,682 (3.5) | 16,555 (6.6) | 0.52** |
| A00-B99 | Certain infectious and parasitic diseases | 13,372 (3.5) | 5,756 (2.3) | 1.53** |
| S00-T98 | Injury, poisoning and certain other consequences of external causes | 10,234 (2.7) | 6,242 (2.5) | 1.07** |
| F00-F99 | Mental and behavioral disorders | 9,726 (2.5) | 12,213 (4.9) | 0.51** |
| E00-E90 | Endocrine, nutritional and metabolic diseases | 9,535 (2.5) | 15,340 (6.1) | 0.39** |
| G00-G99 | Diseases of the nervous system | 7,768 (2.0) | 7,478 (3.0) | 0.67** |
| K00-K93 | Diseases of the digestive system | 7,524 (2.0) | 8,572 (3.4) | 0.56** |
| N00-N99 | Diseases of the genitourinary system | 7,111 (1.8) | 5,984 (2.4) | 0.77** |
| L00-L99 | Diseases of the skin and subcutaneous tissue | 5,038 (1.3) | 5,793 (2.3) | 0.56** |
| C00-D48 | Neoplasms | 4,603 (1.2) | 4,193 (1.7) | 0.71** |
| H60-H95 | Diseases of the ear and mastoid process | 3,418 (0.9) | 2,318 (0.9) | 0.96 |
| H00-H59 | Diseases of the eye and adnexa | 2,852 (0.7) | 1,729 (0.7) | 1.07 |
| Q00-Q99 | Congenital malformations, deformations and chromosomal abnormalities | 1,202 (0.3) | 934 (0.4) | 0.84** |
| D50-D90 | Diseases of the blood and blood-forming organs and certain disorders involving the immune mechanism | 1,063 (0.3) | 2,227 (0.9) | 0.31** |
| O00-O99 | Pregnancy, childbirth and the puerperium | 317 (0.1) | 218 (0.1) | 0.95 |
| V01-Y84 | External causes of morbidity and mortality | 62 (0.0) | 92 (0.0) | 0.44** |
| P00-P96 | Certain conditions originating in the perinatal period | 16 (0.0) | 5 (0.0) | 2.08 |

90,020 patients with 636,057 first visits, i.e., cases without a previous visit to the practice within 14 days, *p < 0.01, **p < 0.001
