# Supplementary material for: Utilization of acute medical services in general practice: a retrospective routine data analysis
Source: Int J Emerg Med. 2025 Aug 7;18:147. doi: 10.1186/s12245-025-00943-y (PMC12333268; doi:10.1186/s12245-025-00943-y)
Supplement: Supplementary file 4 — Supplementary Material 4: Table S4. [file 12245_2025_943_MOESM4_ESM.docx]

**Table S3. Multilevel Regression Results**

|  | | **acute care at first visit** | **course of the episode of care** | | |
| --- | --- | --- | --- | --- | --- |
|  | |  | **same-day** | **3 days** | **2 weeks** |
| model | | Logit (1: acute,  0: elective) | Ordered Logit (1: no additional health service consumption, 2: visit in practice, 3: referral to specialist, 4: hospitalization) | | |
| female | OR | 0.99 | 0.90 | 0.93 | 0.98 |
|  | p-value | (0.2154) | (0.0000) | (0.0000) | (0.0052) |
|  | 95%CI | [0.97,1.01] | [0.88,0.92] | [0.92,0.95] | [0.96,0.99] |
| age (years, reference: *0-6*) | |  |  |  |  |
| *7-17* | OR | 0.52 | 1.40 | 1.57 | 1.69 |
|  | p-value | (0.0000) | (0.0000) | (0.0000) | (0.0000) |
|  | 95%CI | [0.47,0.58] | [1.22,1.62] | [1.42,1.72] | [1.56,1.84] |
| *18-29* | OR | 0.40 | 1.70 | 1.93 | 2.08 |
|  | p-value | (0.0000) | (0.0000) | (0.0000) | (0.0000) |
|  | 95%CI | [0.36,0.44] | [1.49,1.94] | [1.76,2.10] | [1.93,2.25] |
| *30-49* | OR | 0.34 | 1.76 | 1.91 | 2.21 |
|  | p-value | (0.0000) | (0.0000) | (0.0000) | (0.0000) |
|  | 95%CI | [0.36,0.44] | [1.49,1.94] | [1.76,2.10] | [1.93,2.25] |
| *50-64* | OR | 0.27 | 3.15 | 2.93 | 3.49 |
|  | p-value | (0.0000) | (0.0000) | (0.0000) | (0.0000) |
|  | 95%CI | [0.24,0.29] | [2.76,3.59] | [2.69,3.20] | [3.23,3.76] |
| *65-80* | OR | 0.22 | 1.91 | 2.08 | 2.90 |
|  | p-value | (0.0000) | (0.0000) | (0.0000) | (0.0000) |
|  | 95%CI | [0.20,0.24] | [1.67,2.18] | [1.91,2.28] | [2.69,3.14] |
| *>80* | OR | 0.27 | 1.31 | 1.69 | 2.85 |
|  | p-value | (0.0000) | (0.0000) | (0.0000) | (0.0000) |
|  | 95%CI | [0.24,0.29] | [1.14,1.50] | [1.54,1.85] | [2.63,3.09] |
| statutorily insured | OR | 1.60 | 1.46 | 1.40 | 1.41 |
|  | p-value | (0.0000) | (0.0000) | (0.0000) | (0.0000) |
|  | 95%CI | [1.54,1.66] | [1.38,1.55] | [1.33,1.46] | [1.35,1.47] |
| certificate of incapacity for work | OR | 1.93 | 0.52 | 0.64 | 0.82 |
|  | p-value | (0.0000) | (0.0000) | (0.0000) | (0.0000) |
|  | 95%CI | [1.87,1.99] | [0.50,0.54] | [0.62,0.66] | [0.80,0.84] |
| weekdays (reference: Monday) | |  |  |  |  |
| *Tuesday* | OR | 0.57 | 0.91 | 0.86 | 0.93 |
|  | p-value | (0.0000) | (0.0000) | (0.0000) | (0.0000) |
|  | 95%CI | [0.56,0.58] | [0.89,0.93] | [0.84,0.88] | [0.91,0.95] |
| *Wednesday* | OR | 0.60 | 0.80 | 0.71 | 0.85 |
|  | p-value | (0.0000) | (0.0000) | (0.0000) | (0.0000) |
|  | 95%CI | [0.59,0.61] | [0.77,0.82] | [0.69,0.73] | [0.83,0.87] |
| *Thursday* | OR | 0.57 | 0.86 | 0.61 | 0.91 |
|  | p-value | (0.0000) | (0.0000) | (0.0000) | (0.0000) |
|  | 95%CI | [0.56,0.59] | [0.84,0.89] | [0.60,0.63] | [0.89,0.93] |
| *Friday* | OR | 0.59 | 0.79 | 0.66 | 0.89 |
|  | p-value | (0.0000) | (0.0000) | (0.0000) | (0.0000) |
|  | 95%CI | [0.58,0.61] | [0.76,0.81] | [0.65,0.68] | [0.87,0.91] |
| *weekend* | OR | 9.61 | 0.13 | 0.26 | 0.31 |
|  | p-value | (0.0000) | (0.0000) | (0.0000) | (0.0000) |
|  | 95%CI | [8.59,10.75] | [0.11,0.15] | [0.24,0.28] | [0.29,0.33] |
| Diagnoses (ICD-Chapters) | |  |  |  |  |
| *A00-B99: Certain infectious and parasitic diseases* | OR | 1.05 | 1.13 | 1.19 | 1.10 |
|  | p-value | (0.0117) | (0.0001) | (0.0000) | (0.0000) |
|  | 95%CI | [1.01,1.10] | [1.06,1.19] | [1.14,1.25] | [1.06,1.15] |
| *C00-D48: Neoplasms* | OR | 0.91 | 6.13 | 5.14 | 4.51 |
|  | p-value | (0.0021) | (0.0000) | (0.0000) | (0.0000) |
|  | 95%CI | [0.85,0.97] | [5.59,6.72] | [4.69,5.63] | [4.13,4.92] |
| *D50-D90: Diseases of the blood and blood-forming organs* | OR | 0.35 | 1.74 | 1.95 | 2.05 |
|  | p-value | (0.0000) | (0.0000) | (0.0000) | (0.0000) |
|  | 95%CI | [0.31,0.38] | [1.43,2.12] | [1.65,2.30] | [1.77,2.38] |
| *E00-E90: Endocrine, nutritional and metabolic diseases* | OR | 0.48 | 2.10 | 2.10 | 1.89 |
|  | p-value | (0.0000) | (0.0000) | (0.0000) | (0.0000) |
|  | 95%CI | [0.46,0.50] | [1.97,2.23] | [2.00,2.22] | [1.80,1.99] |
| *F00-F99: Mental and behavioral disorders* | OR | 0.44 | 2.79 | 2.32 | 2.19 |
|  | p-value | (0.0000) | (0.0000) | (0.0000) | (0.0000) |
|  | 95%CI | [0.42,0.46] | [2.62,2.96] | [2.19,2.45] | [2.08,2.30] |
| *G00-G99: Diseases of the nervous system* | OR | 0.77 | 1.89 | 1.72 | 1.64 |
|  | p-value | (0.0000) | (0.0000) | (0.0000) | (0.0000) |
|  | 95%CI | [0.74,0.81] | [1.76,2.02] | [1.61,1.83] | [1.55,1.74] |
| *H00-H59: Diseases of the eye and adnexa* | OR | 1.14 | 2.34 | 1.87 | 1.61 |
|  | p-value | (0.0004) | (0.0000) | (0.0000) | (0.0000) |
|  | 95%CI | [1.06,1.23] | [2.12,2.58] | [1.71,2.05] | [1.47,1.75] |
| *H60-H95: Diseases of the ear and mastoid process* | OR | 0.90 | 3.96 | 3.11 | 2.67 |
|  | p-value | (0.0027) | (0.0000) | (0.0000) | (0.0000) |
|  | 95%CI | [0.84,0.96] | [3.67,4.28] | [2.89,3.35] | [2.48,2.87] |
| *I00-I99: Diseases of the circulatory system* | OR | 0.70 | 3.17 | 2.87 | 2.46 |
|  | p-value | (0.0000) | (0.0000) | (0.0000) | (0.0000) |
|  | 95%CI | [0.68,0.73] | [3.02,3.34] | [2.74,3.00] | [2.35,2.57] |
| *J00-J99: Diseases of the respiratory system* | OR | 1.50 | 0.89 | 1.10 | 1.11 |
|  | p-value | (0.0000) | (0.0000) | (0.0000) | (0.0000) |
|  | 95%CI | [1.46,1.55] | [0.85,0.92] | [1.07,1.13] | [1.08,1.14] |
| *K00-K93: Diseases of the digestive system* | OR | 0.55 | 3.93 | 3.41 | 2.99 |
|  | p-value | (0.0000) | (0.0000) | (0.0000) | (0.0000) |
|  | 95%CI | [0.53,0.58] | [3.68,4.19] | [3.22,3.62] | [2.82,3.16] |
| *L00-L99: Diseases of the skin and subcutaneous tissue* | OR | 0.55 | 2.34 | 2.05 | 1.90 |
|  | p-value | (0.0000) | (0.0000) | (0.0000) | (0.0000) |
|  | 95%CI | [0.52,0.57] | [2.16,2.52] | [1.91,2.20] | [1.77,2.03] |
| *M00-M99: Diseases of the musculoskeletal system* | OR | 0.58 | 3.52 | 2.89 | 2.52 |
|  | p-value | (0.0000) | (0.0000) | (0.0000) | (0.0000) |
|  | 95%CI | [0.57,0.60] | [3.41,3.64] | [2.80,2.98] | [2.45,2.60] |
| *N00-N99: Diseases of the genitourinary system* | OR | 0.89 | 2.19 | 1.94 | 1.80 |
|  | p-value | (0.0000) | (0.0000) | (0.0000) | (0.0000) |
|  | 95%CI | [0.85,0.94] | [2.04,2.35] | [1.83,2.07] | [1.70,1.90] |
| *O00-O99: Pregnancy, childbirth and the puerperium* | OR | 0.66 | 2.69 | 2.28 | 1.93 |
|  | p-value | (0.0001) | (0.0000) | (0.0000) | (0.0000) |
|  | 95%CI | [0.54,0.82] | [1.96,3.68] | [1.71,3.05] | [1.42,2.62] |
| *P00-P96: Certain conditions originating in the perinatal period* | OR | 1.36 | 7.10 | 4.36 | 2.82 |
|  | p-value | (0.6116) | (0.0293) | (0.0848) | (0.2724) |
|  | 95%CI | [0.41,4.47] | [1.22,41.41] | [0.82,23.26] | [0.44,18.05] |
| *Q00-Q99: Congenital malformations, deformations* | OR | 0.97 | 1.72 | 1.51 | 1.46 |
|  | p-value | (0.6494) | (0.0000) | (0.0000) | (0.0000) |
|  | 95%CI | [0.87,1.09] | [1.44,2.04] | [1.28,1.78] | [1.25,1.71] |
| *R00-R99: Symptoms, signs and abnormal clinical and laboratory* | OR | 0.54 | 2.14 | 1.92 | 1.75 |
|  | p-value | (0.0000) | (0.0000) | (0.0000) | (0.0000) |
|  | 95%CI | [0.53,0.56] | [2.06,2.21] | [1.87,1.98] | [1.70,1.79] |
| *S00-T98: Injury, poisoning and other consequences of external* | OR | 1.01 | 4.12 | 3.45 | 2.95 |
|  | p-value | (0.6983) | (0.0000) | (0.0000) | (0.0000) |
|  | 95%CI | [0.97,1.05] | [3.89,4.37] | [3.27,3.64] | [2.80,3.10] |
| *V01-Y84: External causes of morbidity and mortality* | OR | 0.57 | 1.18 | 1.23 | 1.46 |
|  | p-value | (0.0091) | (0.7027) | (0.5931) | (0.2834) |
|  | 95%CI | [0.37,0.87] | [0.50,2.81] | [0.57,2.67] | [0.73,2.91] |
| *Z00-Z99: Factors influencing health status and contact with health services* | OR | 0.60 | 0.26 | 0.37 | 0.39 |
|  | p-value | (0.0000) | (0.0000) | (0.0000) | (0.0000) |
|  | 95%CI | [0.59,0.61] | [0.25,0.27] | [0.36,0.38] | [0.38,0.40] |
| *U00-U99: Codes for special purposes* | OR | 1.25 | 1.30 | 2.16 | 2.01 |
|  | p-value | (0.0000) | (0.0000) | (0.0000) | (0.0000) |
|  | 95%CI | [1.20,1.29] | [1.23,1.36] | [2.09,2.23] | [1.95,2.06] |
| Practice fixed effects | | *yes* | *yes* | *yes* | *yes* |
| Patient random effects | | *yes* | *yes* | *yes* | *yes* |
| observations | | 636,016 | 385,446 | 385,446 | 385,446 |
| patients | | 90,015 | 83,626 | 83,626 | 83,626 |

OR: Odds Ratio; individual practice and patient level effects; *p*-values in parentheses; 95% confidence intervals in brackets
