# Supplementary material for: Utilization of acute medical services in general practice: a retrospective routine data analysis
Source: Int J Emerg Med. 2025 Aug 7;18:147. doi: 10.1186/s12245-025-00943-y (PMC12333268; doi:10.1186/s12245-025-00943-y)
Supplement: Supplementary file 5 — Supplementary Material 5: STROBE Statement - checklist of items that should be included in reports of observational studies. [file 12245_2025_943_MOESM5_ESM.docx]

**Appendix 2.** STROBE Statement—checklist of items that should be included in reports of observational studies

|  | Item No. | Recommendation | Page  No. | Relevant text from manuscript |
| --- | --- | --- | --- | --- |
| **Title and abstract** | 1 | (*a*) Indicate the study’s design with a commonly used term in the title or the abstract | p.1, p.2 | A Retrospective Routine Data Analysis  The retrospective cohort study used routine data |
|  |  | (*b*) Provide in the abstract an informative and balanced summary of what was done and what was found | p.2 | Methods & Results |
| Introduction | | | |  |
| Background/rationale | 2 | Explain the scientific background and rationale for the investigation being reported | p.3-4 |  |
| Objectives | 3 | State specific objectives, including any prespecified hypotheses | p.4 | The objective of the present study is to quantify and describe … |
| Methods | | | |  |
| Study design | 4 | Present key elements of study design early in the paper | p.4-5 | In this retrospective cohort study,… |
| Setting | 5 | Describe the setting, locations, and relevant dates, including periods of recruitment, exposure, follow-up, and data collection | p.4-5 |  |
| Participants | 6 | (*a*) *Cohort study*—Give the eligibility criteria, and the sources and methods of selection of participants. Describe methods of follow-up  *Case-control study*—Give the eligibility criteria, and the sources and methods of case ascertainment and control selection. Give the rationale for the choice of cases and controls  *Cross-sectional study*—Give the eligibility criteria, and the sources and methods of selection of participants | p.4-5 |  |
|  |  | (*b*) *Cohort study*—For matched studies, give matching criteria and number of exposed and unexposed  *Case-control study*—For matched studies, give matching criteria and the number of controls per case | NA | NA |
| Variables | 7 | Clearly define all outcomes, exposures, predictors, potential confounders, and effect modifiers. Give diagnostic criteria, if applicable | p.6 |  |
| Data sources/ measurement | 8* | For each variable of interest, give sources of data and details of methods of assessment (measurement). Describe comparability of assessment methods if there is more than one group | p.5-7 |  |
| Bias | 9 | Describe any efforts to address potential sources of bias | p.7 |  |
| Study size | 10 | Explain how the study size was arrived at | p.4-5 | All patients with at least one documented consultation in one of the participating practices during the study period (2022-2023) were included in the analysis,… |

Continued on next page

| Quantitative variables | 11 | Explain how quantitative variables were handled in the analyses. If applicable, describe which groupings were chosen and why | p.7 |  |
| --- | --- | --- | --- | --- |
| Statistical methods | 12 | (*a*) Describe all statistical methods, including those used to control for confounding | p.7 |  |
|  |  | (*b*) Describe any methods used to examine subgroups and interactions | p.7 |  |
|  |  | (*c*) Explain how missing data were addressed | p.7 | In the bivariate analysis, missing values were deleted of casewise, while in the multivariate analysis, they were deleted listwise. |
|  |  | (*d*) *Cohort study*—If applicable, explain how loss to follow-up was addressed  *Case-control study*—If applicable, explain how matching of cases and controls was addressed  *Cross-sectional study*—If applicable, describe analytical methods taking account of sampling strategy | NA | NA |
|  |  | (*e*) Describe any sensitivity analyses | p.6 | As our definition of acute cases might lead to a rather liberal definition of acuteness, control visits after the initial visit were also taken into account. Therefore, a second (conservative) definition is applied by … |
| Results | | | | |
| Participants | 13* | (a) Report numbers of individuals at each stage of study—eg numbers potentially eligible, examined for eligibility, confirmed eligible, included in the study, completing follow-up, and analysed |  | Table 1 |
|  |  | (b) Give reasons for non-participation at each stage |  | NA |
|  |  | (c) Consider use of a flow diagram |  | NA |
| Descriptive data | 14* | (a) Give characteristics of study participants (eg demographic, clinical, social) and information on exposures and potential confounders |  | Table 1 |
|  |  | (b) Indicate number of participants with missing data for each variable of interest |  | Table 1, Table 3 |
|  |  | (c) *Cohort study*—Summarise follow-up time (eg, average and total amount) | NA | NA |
| Outcome data | 15* | *Cohort study*—Report numbers of outcome events or summary measures over time |  | Tables 1-2 |
|  |  | *Case-control study—*Report numbers in each exposure category, or summary measures of exposure |  |  |
|  |  | *Cross-sectional study—*Report numbers of outcome events or summary measures |  |  |
| Main results | 16 | (*a*) Give unadjusted estimates and, if applicable, confounder-adjusted estimates and their precision (eg, 95% confidence interval). Make clear which confounders were adjusted for and why they were included |  | Table 1 |
|  |  | (*b*) Report category boundaries when continuous variables were categorized |  | NA |
|  |  | (*c*) If relevant, consider translating estimates of relative risk into absolute risk for a meaningful time period |  | NA |

Continued on next page

| Other analyses | 17 | Report other analyses done—eg analyses of subgroups and interactions, and sensitivity analyses |  | Table 1, Table S1, Table S2, Table S3 and Table S4 |
| --- | --- | --- | --- | --- |
| Discussion | | | | |
| Key results | 18 | Summarise key results with reference to study objectives | p.12 | Our data of over 870,000 consultations reveal that at least 60% of all primary care consultations are for acute care needs. |
| Limitations | 19 | Discuss limitations of the study, taking into account sources of potential bias or imprecision. Discuss both direction and magnitude of any potential bias | p.14-15 |  |
| Interpretation | 20 | Give a cautious overall interpretation of results considering objectives, limitations, multiplicity of analyses, results from similar studies, and other relevant evidence | p.16 |  |
| Generalisability | 21 | Discuss the generalisability (external validity) of the study results | p.13-14 |  |
| Other information | |  | | |
| Funding | 22 | Give the source of funding and the role of the funders for the present study and, if applicable, for the original study on which the present article is based | p.17 | The study was funded by the Central Research Institute of Ambulatory Health Care in Germany (Zentralinstitut für die kassenärztliche Versorgung, Zi). The study was conducted independently. |

*Give information separately for cases and controls in case-control studies and, if applicable, for exposed and unexposed groups in cohort and cross-sectional studies.
